# Embedding Rehabilitation as Core Cancer Care in Australia and New Zealand: A Health System Imperative

**DOI:** 10.5694/mja2.70226

**Published:** 2026-06-09

**Authors:** Krystal Song, Steven G. Faux, Fary Khan

**Affiliations:** ^1^ Department of Rehabilitation The Royal Melbourne Hospital Melbourne Victoria Australia; ^2^ Department of Medicine The University of Melbourne Melbourne Victoria Australia; ^3^ Department of Rehabilitation Medicine St Vincent's Hospital Darlinghurst New South Wales Australia; ^4^ Faculty of Medicine University of New South Wales Sydney New South Wales Australia; ^5^ Qatar Rehabilitation Institute Hamad Bin Khalifa University Doha Qatar

**Keywords:** cancer rehabilitation, cancer survivorship, interdisciplinary rehabilitation

## Abstract

Cancer remains a global health challenge, with rising survivorship rates highlighting the need for integrated interdisciplinary rehabilitation care. Survivors frequently experience persistent physical, functional, psychological, cognitive and behavioural challenges, including fatigue, deconditioning, neuropathy, pain and psychological distress, with up to two‐thirds reporting significant unmet needs and reduced quality of life. Interdisciplinary rehabilitation, encompassing exercise, education, nutrition, task‐specific functional retraining, psychosocial support and vocational interventions, effectively mitigates these disabilities, improving function and promoting societal participation. Despite strong evidence, rehabilitation remains underutilised in Australia and New Zealand due to workforce, infrastructure, referral, funding and awareness barriers. Embedding rehabilitation as standard cancer care is essential to optimise survivorship outcomes and deliver sustainable health system benefits.

## 1 Introduction

Cancer remains a major global health challenge. In 2022, close to 20 million new cases were diagnosed worldwide, with almost 10 million cancer‐related deaths recorded [1]. In Australia and New Zealand, more than 250,000 new cancer diagnoses and over 63,000 cancer‐related deaths were recorded that year, with breast, prostate and colorectal cancers among the most commonly diagnosed cancers [1]. Current advances in screening, diagnosis and treatments have substantially improved survival. In Australia, the age‐standardised 5‐year relative survival of all cancers combined was 70.6% in 2014–2019, with further improvement anticipated in the coming years [2]. As survival improves, the number of people living with and beyond cancer continues to grow. In Australia, it is projected that nearly 1.9 million people will be cancer survivors by 2040 [2]. This shift necessitates comprehensive, coordinated rehabilitation across the cancer continuum to ensure sustainable survivorship care.

## 2 Cancer Survivorship and Rehabilitation Needs

Life after a cancer diagnosis is frequently accompanied by ongoing health challenges. Many patients experience persistent physical, functional, psychological, cognitive and/or behavioural effects from the cancer and related treatments [3]. Deconditioning, cancer‐related fatigue, peripheral neuropathy, pain, cardiotoxicity, sarcopenia, psychological distress and cognitive impairment are common, and up to 60%–70% of cancer survivors report significant functional limitations and unmet supportive care needs [4, 5]. These challenges are associated with personal, social and economic consequences, along with diminished quality of life (QoL), disrupted participation in work and societal roles [6]. Complementing mortality and morbidity outcomes, functioning is recognised by the World Health Organization (WHO) as the third key indicator of health [7]. Although oncological advances have improved survival rates, many cancer survivors experience complex and often prolonged disability, underscoring the importance of optimising functioning as a core outcome of cancer care [5]. In addition, cancer represents a substantial economic burden, accounting for the highest disease‐specific health expenditure in Australia in 2023–2024, estimated at $19.7 billion (10.9% of total health expenditure) [2]. Without systematic strategies to identify and address functional morbidity, health systems will face escalating survivorship‐related disability and healthcare utilisation costs [2, 6].

This challenge presents a timely opportunity for reform. In 2023, the WHO World Health Assembly endorsed a landmark resolution recognising rehabilitation as an essential component of healthcare within existing health systems and across the life course. As signatories, Australia and New Zealand are therefore well positioned and mandated to embed rehabilitation within routine cancer care as part of sustainable, high‐quality service delivery. Globally, this agenda is advanced by the World Rehabilitation Alliance, in collaboration with the International Society of Physical and Rehabilitation Medicine (ISPRM), International Psycho‐Oncology Society, World Confederation for Physical Therapy, World Federation of Occupational Therapists and the International Spinal Cord Society (ISCoS), working collectively to promote rehabilitation as a system‐level priority [8]. Within this policy context, cancer rehabilitation provides a practical framework for addressing the complex and multifaceted functional morbidities faced by many cancer survivors.

Cancer rehabilitation is a key component of comprehensive cancer care, enabling timely assessment of patient needs and delivery of targeted, evidence‐based interventions across the disease trajectory [9]. Its goals are to mitigate treatment‐related toxicities, optimise functional independence, support participation in meaningful life roles and improve psychological well‐being and QoL [9]. Cancer rehabilitation may be delivered as uni‐disciplinary care or coordinated interdisciplinary programmes that are individualised and goal‐directed. Rehabilitation interventions can be delivered across the cancer continuum, from prehabilitation before treatment and active treatment, through to survivorship and palliative care phases, and across various settings (including inpatient, outpatient, community and home‐based services) [10, 11, 12, 13, 14, 15, 16]. Core components include patient education (including symptom self‐management, energy conservation and lifestyle modification strategies); physical reconditioning and structured exercise; task‐specific functional retraining to improve independence within activities of daily living; nutritional optimisation; psychosocial interventions (including cognitive behavioural therapy, mindfulness and supportive counselling); fatigue and sleep management; social, vocational and return‐to‐driving programme support; and specialised therapies, such as lymphoedema management, vestibular rehabilitation and pelvic floor therapy [9].

The evidence base for cancer rehabilitation is expanding, with randomised controlled trials and systematic reviews across diverse cancer populations and care settings demonstrating improvements in functional capacity, cancer‐related fatigue, psychological outcomes and QoL [10, 11, 12, 13, 14, 15, 16]. Multicomponent interventions have been evaluated in common solid tumours (including breast, lung, colorectal, brain and prostate cancers) and haematological malignancies, spanning prehabilitation, rehabilitation during active treatment and post‐treatment survivorship [10, 11, 12, 13, 14, 15, 16]. The strength of evidence varies by cancer type, intervention and care settings. In breast cancer, exercise, lymphoedema management and psychosocial interventions following treatment improve symptoms and QoL, although programme content and study quality are heterogenous [10]. Exercise training improves functional capacity and health‐related QoL following lung cancer resection [11]. In colorectal cancer, moderate‐ to high‐quality evidence supports exercise and multimodal prehabilitation for improving physical fitness and perioperative outcomes [12]. Multidisciplinary rehabilitation in ambulatory settings is associated with sustained functional gains in brain tumour survivors [13]. Evidence in haematological malignancies is emerging but remains less extensive than for common solid tumours [15]. In addition, inpatient, ambulatory and home‐based rehabilitation models have demonstrated clinically meaningful benefits in function and QoL [15, 16]. Given the multidimensional nature of cancer‐related disability, exercise‐only interventions are unlikely to address the full spectrum of cancer survivor needs and coordinated, comprehensive models of rehabilitation care are required.

## 3 Barriers and the Way Forward

Despite a growing evidence base, and national frameworks from the Clinical Oncology Society of Australia (COSA) and Cancer Council Australia highlighting survivorship care, uptake of cancer rehabilitation within routine oncology care remains low across Australia and New Zealand [17, 18]. Barriers to broader uptake are multifactorial and include workforce capacity limitations, inadequate infrastructure, fragmented referral pathways, funding constraints and variable clinician awareness (Table 1) [17, 18]. Sustainable funding for staffing, capacity building, infrastructure and programme delivery remains inadequate, limiting ongoing provision of services [17, 18]. Limited awareness among oncologists, nurses, general practitioners and patients regarding the scope, benefits and availability of rehabilitation services further perpetuates low referral and uptake [17]. Access to rehabilitation services varies across jurisdictions and sectors, with rural and remote communities disproportionately affected [18]. Fragmentation between oncology, primary care and rehabilitation services results in poor information sharing, missed or delayed referrals and reduced coordination of care [19]. Although interdisciplinary rehabilitation programmes exist across public and private care settings and are well positioned to support patients living with and beyond cancer, they remain underutilised and often difficult to access. Public sector programmes typically prioritise patients with significant functional impairment and medical complexity, and are constrained by capacity and eligibility thresholds, while private services offer earlier access for patients with private health insurance or capacity to self‐fund, contributing to inequities in access. Fewer than 20%–30% of patients with cancer‐related functional impairment are referred for rehabilitation despite substantially higher unmet needs [9]. Referral often depends on individual clinician awareness rather than systematic screening or standardised referral pathways. Many cancer survivors instead access fragmented, time‐limited allied health services through fee‐for‐service models, with limited subsidies and significant out‐of‐pocket costs restricting continuity and coordinated care.

**TABLE 1:** Key issues, barriers to implementation and priority actions to strengthen cancer rehabilitation.

Issues	Barriers to implementation	Priority actions	Actors
Embedding rehabilitation within cancer care	Limited awareness of cancer rehabilitation and its benefits among patients and clinicians Rehabilitation is not routinely integrated within oncology care pathways	Establish in‐reach rehabilitation services within oncology settings Integrate rehabilitation medicine physicians within cancer multidisciplinary team forums Develop clear referral pathways to rehabilitation medicine services Provide education on cancer rehabilitation for oncology clinicians and referrers Support integrated oncology‐rehabilitation service models and telerehabilitation programmes	Health services Clinical workforce: oncology, surgical and rehabilitation clinicians Professional organisations: AFRM, RACP, RMSANZ, COSA Consumer organisations
Recognition of function as a key outcome of cancer care	Functional outcomes are not routinely assessed in oncology practice Lack of standardised functional screening tools within oncology workflows	Implement routine functional screening across the cancer care continuum Embed validated functional outcome measures within electronic medical records and cancer registries	Health services National cancer agencies: Cancer Australia, COSA Clinical registries: AROC and other quality registries
Limited access to prehabilitation and survivorship rehabilitation	Limited implementation of structured prehabilitation programmes and community‐based rehabilitation services	Develop national evidence‐based guidelines for cancer prehabilitation and rehabilitation Expand rehabilitation services across the cancer care continuum, including rehabilitation‐in‐the‐home models	Health services Professional organisations: AFRM, RACP, RMSANZ, COSA Cancer organisations: Cancer Council Australia Research funding bodies
Underutilisation of rehabilitation entitlements in private health insurance	Uncertainty regarding eligibility for Hospital in the Home rehabilitation services Limited awareness of referral pathways to private sectors	Review *Private Health Insurance Act* provisions relating to Hospital in the Home rehabilitation services Improve referral pathways to rehabilitation services across primary, secondary and private healthcare settings	Government: Department of Health, Disability and Ageing Private health sector: PHA, APHA, insurers Professional organisations: AFRM, RACP, RMSANZ, COSA Health services Consumer organisations: CHF, HCCA
Inequitable access for rural and underserved populations	Geographic barriers and fragmented coordination of rehabilitation services across the cancer care continuum	Develop consumer co‐designed pathways to improve equitable access Expand telehealth and outreach cancer rehabilitation programmes in rural and regional areas	State and territory governments Health services Consumer organisations
Limited data and research to guide cancer rehabilitation services	National datasets underutilised in capturing functional outcomes and rehabilitation service utilisation in cancer survivors	Use of nationally endorsed minimum datasets for functional screening and rehabilitation outcomes Invest in health services and implementation research to inform cancer rehabilitation models of care	Cancer organisations: Cancer Council Australia, COSA Research funding bodies Academic and clinical research groups Clinical registries: AROC

Abbreviations: AFRM, Australasian Faculty of Rehabilitation Medicine; APHA, Australian Private Hospitals Association; AROC, Australasian Rehabilitation Outcomes Centre; CHF, Consumers Health Forum of Australia; COSA, Clinical Oncology Society of Australia; HCCA, Health Care Consumers' Association; PHA, Private Healthcare Australia; RACP, Royal Australasian College of Physicians; RMSANZ, Rehabilitation Medicine Society of Australia and New Zealand.

Although survivorship is improving in many countries, the capacity of health systems to reduce cancer‐related disability has not kept pace. Cancer care remains predominantly treatment‐ and surveillance‐focused, with functional outcomes rarely incorporated as system performance indicators [18]. Embedding rehabilitation as a core component of cancer care requires coordinated action across the six WHO health system building blocks, including workforce capacity building, service delivery models, health information systems, infrastructure, financing and governance [20]. Priorities to help overcome barriers to cancer rehabilitation implementation (Table 1) include strengthening workforce education and capacity across oncology, surgery and primary care to improve recognition of functional concerns and support early referral. Integrating rehabilitation physicians into cancer interdisciplinary teams may enable timely identification of needs and coordinated care planning. Development of structured post‐operative referral pathways and early in‐reach rehabilitation review can further support proactive management of functional morbidity. Leveraging digital infrastructure can support shared care plans, service directories and electronic referral pathways [19] and help improve coordination between oncology, rehabilitation and primary care services. Embedding validated functional screening tools and automated referral prompts within electronic medical records may enable earlier identification of rehabilitation needs [19]. Structured prehabilitation is increasingly incorporated into major cancer surgery pathways, consistent with published perioperative care frameworks and standards [21]. Expansion of ambulatory rehabilitation capacity through group‐based community programmes and flexible rehabilitation‐in‐the‐home models could further improve access and continuity of care [15]. Policy reform in the private sector is also needed to recognise and support access to rehabilitation during and after cancer treatment. Government review of private hospital funding and legislative changes to Hospital in the Home provisions under the Commonwealth *Private Health Insurance Act* 2007 may improve access to flexible rehabilitation models. National clinical guidelines jointly endorsed by oncology and rehabilitation bodies would provide critical structure and legitimacy. Innovative models of care such as integrated oncology‐rehabilitation clinics, telerehabilitation, e‐health technology use, rural community‐based programmes, models of peer support, transport initiatives and multimodal return‐to‐work interventions offer scalable approaches to address financial and geographical constraints but require further evaluation and policy support [18].

The growing burden of treatment‐related disability is a predictable consequence of modern cancer care. Rehabilitation should be integrated as a routine component of high‐quality oncology, not an optional adjunct. Transforming outcomes for cancer survivors will require collaborative actions across the health system, including policymakers, clinicians, researchers, funders, consumer organisations and non‐government advocacy groups, to advance the agenda of cancer rehabilitation and ensure equitable access to rehabilitation for all cancer survivors [18]. Investment in health services and systems research is needed to better define current patterns of service use, gaps in care and optimal models of rehabilitation delivery across Australia and New Zealand. Development of integrated survivorship pathways linking oncology, rehabilitation and primary care, supported by telehealth and digital solutions will be critical. The utilisation of a nationally endorsed minimum dataset for functional screening and outcomes through the Australasian Rehabilitation Outcomes Centre would enable benchmarking, guide resource allocation and help reduce inequities in survivorship care [22]. Clinicians should incorporate routine functional screening and early referral into standard cancer care pathways [23]. Health services should embed interdisciplinary rehabilitation within models of cancer care, expand dedicated cancer rehabilitation services across public and private health settings, and strengthen coordination across inpatient, ambulatory and community settings [24]. Professional colleges and cancer organisations should facilitate workforce development and core competency standards. Funders and policymakers should align financing and service planning to ensure timely access to coordinated rehabilitation across the care continuum, with targeted strategies to address the needs of rural, socio‐economically disadvantaged and underserved populations. Consumer and advocacy organisations have a critical role in raising awareness, shaping service co‐design and ensuring that survivorship priorities are reflected in policy and funding decisions [25]. Examples of practical steps to support the establishment of cancer rehabilitation services are outlined in Box 1.

BOX 1 **Practical steps to establish cancer rehabilitation services.**

**Table:** 

Clinicians and health services seeking to develop cancer rehabilitation services may consider the following initial steps: Engage local rehabilitation services: Contact rehabilitation physicians within your local department of rehabilitation medicine to identify existing services that may support patients with cancer, including outpatient clinics, ambulatory or community‐based programmes. Establish dedicated referral pathways: Develop clear referral pathways from oncology and surgical services to rehabilitation medicine services, including outpatient, ambulatory and community‐based programmes, to facilitate early identification and management of functional concerns. Develop dedicated cancer rehabilitation services: Establish dedicated rehabilitation clinics or integrated models of care within existing rehabilitation services to provide coordinated assessment and management of cancer‐related impairments. Strengthen workforce capability: Engage professional organisations such as AFRM and the RMSANZ to support development of education modules and training resources in cancer rehabilitation. International resources from the ISPRM Cancer Rehabilitation Special Interest Group may also support workforce capability and expertise. Implement routine functional screening and assessment: Incorporate validated functional and patient‐reported outcome measures into routine care to identify rehabilitation needs, monitor outcomes and support timely referral. Commonly used measures include the PROMIS‐29 profile [25], ClinFit [19] or other validated functional outcome tools appropriate to the clinical setting. Collaborate with oncology teams: Involve rehabilitation clinicians in multidisciplinary cancer meetings, survivorship programmes and care planning processes to facilitate early recognition of rehabilitation needs and coordinated survivorship care planning.

Abbreviations: AFRM, Australasian Faculty of Rehabilitation Medicine; ClinFit, Clinical Functioning Information Tool [19]; ISPRM, International Society of Physical and Rehabilitation Medicine; PROMIS‐29, Patient‐Reported Outcomes Measurement Information System [25]; RMSANZ, Rehabilitation Medicine Society of Australia and New Zealand.

## 4 Conclusion

Without systematic integration of rehabilitation within cancer care, many cancer survivors will continue to experience avoidable functional decline, reduced participation and increased healthcare utilisation. Embedding rehabilitation within routine cancer care represents a practical opportunity to improve long‐term outcomes, strengthen system sustainability and promote more equitable survivorship care across Australia and New Zealand [18].

## Author Contributions

**Krystal Song:** conceptualisation, writing – original draft, writing – editing and review, approval of final manuscript. **Steven G. Faux:** writing – editing and review, approval of final manuscript. **Fary Khan:** writing – editing and review, approval of final manuscript.

## Funding

The authors have nothing to report.

## Disclosure

Not commissioned; externally peer reviewed.

## Conflicts of Interest

The authors declare no conflicts of interest.

## Data Availability

The authors have nothing to report.
